# A Value-Based Medicine cost-utility analysis of genetic testing for neovascular macular degeneration

**DOI:** 10.1186/s40942-015-0016-5

**Published:** 2015-10-26

**Authors:** Gary C. Brown, Melissa M. Brown, Heidi B. Lieske, Philip A. Lieske, Kathryn S. Brown

**Affiliations:** 1 Center for Value-Based Medicine®, 6010 West Mill Road, Flourtown, PA 19031 USA; 2 The Eye Research Institute, Philadelphia, PA USA; 3 The Retina Service, Wills Eye Hospital, Jefferson Medical University, Philadelphia, PA USA; 4 The Research Department, Wills Eye Hospital, Jefferson Medical University, Philadelphia, PA USA

**Keywords:** Genetic testing, Neovascular macular degeneration, Comparative effectiveness, Cost-effectiveness

## Abstract

**Background:**

There is a dearth of patient, preference-based cost-effectiveness analyses evaluating genetic testing for neovascular age-related macular degeneration (NVAMD).

**Methods:**

A Value-Based Medicine, 12-year, combined-eye model, cost-utility analysis evaluated genetic testing of Category 3 AMD patients at age 65 for progression to NVAMD. The benefit of genetic testing was predicated upon the fact that early-treatment ranibizumab therapy (baseline vision 20/40–20/80) for NVAMD confers greater patient value than late-treatment (baseline vision ≤20/160). Published genetic data and MARINA Study ranibizumab therapy data were utilized in the analysis. Patient value (quality-of-life gain) and financial value (2012 US real dollar) outcomes were discounted at 3 % annually.

**Results:**

Genetic testing-enabled, early-treatment ranibizumab therapy per patient conferred mean 20/40^−1^ vision, a 0.845 QALY gain and 14.1 % quality-of-life gain over sham therapy. Late-treatment ranibizumab therapy conferred mean 20/160^+2^ vision, a 0.250 QALY gain and 4.2 % quality-of-life gain over sham therapy. The gain from early-treatment over late-treatment was 0.595 QALY (10.0 % quality-of-life gain). The per-patient cost for genetic testing/closer monitoring was $2205 per screened person, $2.082 billion for the 944,000 estimated new Category 3 AMD patients annually. Genetic testing/monitoring costs per early-treatment patient totaled $66,180. Costs per early-treatment patient included: genetic testing costs: $66,180 + direct non-ophthalmic medical costs: −$40,914 + caregiver costs: −$172,443 + employment costs: −$14,098 = a net societal cost saving of $160,582 per early treatment patient. When genetic screening facilitated an incremental 12,965 (8.0 %) of the 161,754, new annual NVAMD patients aged ≥65 in the US to undergo early-treatment ranibizumab therapy, each additional patient treated accrued an overall, net financial gain for society of $160,582. Genetic screening was cost-effective, using World Health Organization criteria, when it enabled an incremental 4.1 % (6634) of 161,754 annual NVAMD patients ≥65 years to receive early-treatment ranibizumab therapy.

**Conclusions:**

Genetic screening-enabled, early-treatment ranibizumab therapy for NVAMD is cost-effective if it enables an incremental 4.1 % of the annual US cohort of new-onset NVAMD patients ≥65 to undergo early-treatment with ranibizumab.

## Background

Age-related macular degeneration (AMD) is the leading cause of blindness in the ≥60-year-old population in the US [1]. Eye Diseases Prevalence Research Group [2] data suggest advanced AMD (central geographic atrophy and/or neovascular AMD) affected 1.87 million people in 2012 [3]. Among advanced AMD patients, 70 % had neovascular AMD (NVAMD) in one or both eyes [2]. Approximately 171,350 patients developed NVAMD annually [2, 3]. Among US citizens reaching age 65 annually, 944,400 have Category 3 drusen [2, 3].

Several models predict the conversion of atrophic AMD to NVAMD, each using the Age Related Eye Diseases Study (AREDS) classification of AMD [4–8]. The more complex model in AREDS Report No. 17 [6], evaluated the eyes of 3212 participants, utilizing drusen severity and pigmentary abnormalities at baseline. The authors proposed a 9-step severity scale that combined a 6-step drusen area scale with a 5-step pigmentary abnormality scale. The 5-year risk of progression to advanced AMD varied from <1 % in Steps 1 and 2 to 43.5 % in Step 9 [6]. Nonetheless, the scale more accurately predicts central geographic atrophy (43.5 % in Step 9) than NVAMD (4.8 % in Step 9, but 21.1 % in the less severe Step 8) [6]. A simplified severity scale in AREDS Report No. 18 used large drusen and pigmentary changes in a 0–4 scale and demonstrated, when both were present bilaterally, the 5-year incidence of advanced AMD in at least one eye was 47.3 % [7]. While a top score of 4 identified 67.8 % of 5-year progressors to advanced AMD, it identified only 36.5 % progressing to NVAMD [7].

Blue Mountains Eye Study investigators [8], using an AREDS simplified severity scale, found a generalized estimating equation model showed 61.5 % of patients with bilateral drusen ≥125 μm and bilateral retinal pigment epithelial changes progressed to advanced AMD over 10 years. Assuming 70 % of those advanced AMD cases were neovascular [2], approximately 43 % of NVAMD cases would be identified. This converts to (57.0 % × 161,754 cases=) 92,200 new NVAMD patients aged ≥65 in the US not identified by phenotypic markers annually [1–3].

Multiple advances in AMD genetics have been made over the last decade [9-17]. A report by Seddon et al. [12], created models predicting advanced AMD development. They approach include age, gender, education, smoking, body mass index, single nucleotide polymorphisms in the CFH, ARMS2/HTRA1, C3, C2, and CFB genes, as well as important markers discovered through two large genome-wide association studies [4, 13–16]. With the inclusion of cholesterol metabolic markers CETP, LIPC and ABCA1, the progression prediction to advanced disease within 10 years achieved unparalleled accuracy (C = 0.90) [4]. Using this model for a dichotomous risk score (“risk” vs “non risk”), Yu et al. [4], showed the 5-year progression of Category 3 AMD cases to advanced AMD could be predicted with both sensitivity and specificity over 80 %; sensitivity over 10 years was ≥90 %. There was a 10-year 20 % progression to neovascular AMD (Table 1) using phenotypic and genotypic markers, but they identified 90 % of people progressing to neovascular AMD [4].

**Table 1:** Progression for 2560 AREDS AMD (Category 1–3 at Baseline) patients over 10.3 years (Yu et al. [4])

AMD category	n	Progressed to next stage	No progression
1	713	494 (69 %)	219 (31 %)
2	1190	376 (32 %)	814 (68 %)
3	1527	578 (8 %)	949 (62 %)
3–4	280	280 (18 %)	NA
3–5	298	298 (20 %)	NA

Includes patients treated and not treated with AREDS supplements [5]

*AREDS* Age-Related Eye Disease Study, *AMD* age-related macular degeneration, *NA* not applicable

### Neovascular AMD therapy

Intravitreal ranibizumab therapy for NVAMD is among the greatest medical advances over the decade [18–24]. Earlier therapy has a better visual prognosis than later therapy [20]. Thus, it is hoped greater risk awareness will allow patients to seek earlier care.

Considering the importance of earlier ranibizumab therapy, the authors undertook a Value-Based Medicine^®^ (VBM) [22–24], societal, cost-utility analysis to assess the patient preference-based, comparative effectiveness and cost-effectiveness (cost-utility) of genetic testing for NVAMD.

## Methods

Features associated with genetic testing for NVAMD, and the economic modeling assumptions used are listed in Table 2. Comparative effectiveness quantified the incremental *patient value gain* (improvement in quality-of-life and/or length-of-life), though length-of-life change was not included, since better vision has not been well shown to lengthen life. Outcomes were measured in: (1) percent value gain, and (2) quality-adjusted life-year (QALY) gain [18, 20, 22–25]. QALY gain was calculated by multiplying: (utility gain) × (years of interventional benefit). Financial metrics include: (1) cost-utility ratio, $/QALY, or dollars expended per QALY gained, associated with genetic testing-enabled, early-treatment ranibizumab for NVAMD, and (2) societal costs.

**Table 2:** Genetic testing for neovascular AMD. Cost-utility analysis model parameters and assumptions

Phenotypic Features			
Identification of 5-year progression to advanced AMD (age-related macular degeneration) using the AREDS (Age-Related Eye Disease Study) Simplified Scale for AMD [7] Identified 67.8 % of progressors to atrophic AMD, but only 36.5 % of those progressing to neovascular AMD			

Genotypic Features (from Yu et al. [4])			
SNPs in the following genes			
Gene [4]	Hazard ratio [4] for neovascular AMD	p-value	Notes
CFH—3 SNP	1.37	2.2 × 10^−4^	Higher risk
ARMS2/HTRA1—2 SNPs	1.27	5.3 × 10^−3^	Higher risk
C2	0.60	0.14	Uncertain status
C3	1.25	0.02	Higher risk
CFB	0.57*	0.02	Lower risk
LIPC	0.57*	0.04	Lower risk
CFI	0.87	0.10	Uncertain status
TIMP3	0.79	0.30	Uncertain status
CETP	1.17	0.06	Uncertain status
ABCA1	0.97	0.79	Uncertain status
COLSA1	1.2	0.21	Uncertain status
APOE—2 SNPs	0.97	0.85	Uncertain status
Genetic profiles			
High-risk = homozygous on all genetic loci for the alleles that increase the risk of advanced AMD			
Medium-risk = heterozygous on genetic loci for the alleles that increase/decrease the risk of advanced AMD			
Low-risk = homozygous on all genetic loci for alleles that decrease the risk of advanced AMD			
As per Yu et al. [4], all genes listed above are assumed to be tested for. This conservative assumption likely biases against the financial value gain and cost-effectiveness by increasing what will likely be decreased genetic costs in the future			
Progression to neovascular AMD			
Incremental, 10-year progression rate to neovascular AMD for Category 3 AMD patients with: a past smoking history, BMI of 25–29, normal fellow eye and greater than a high school education [4]			

Genetic profile	10-year progression	Percent of progressors detected
High-risk	26%	90%
Medium-risk	3%	9%
Low-risk	<1%	1%
Among people with Category 3 AMD, 20 % will progress to neovascular AMD over 10 years [4]. (SNP = single nucleotide polymorphism, * = decreased chance of progression to neovascular AMD, BMI = basal metabolic index)		
Neovascular AMD therapy		
Clinical features, MARINA study [18, 20, 23]		
All participants had minimally classic or occult, subfoveal choroidal neovascularization		
Baseline vision in the affected eye: 20/40–20/320		
Choroidal neovascular lesions <12 disc areas at baseline		
Mean baseline vision: 20/80-1 in the ranibizumab treatment and sham therapy cohorts		
Mean baseline age: 75 years		
Participants randomized 1:1:1 to: (1) 0.5 mg intravitreal ranibizumab dose (n = 240), (2) 0.3 mg intravitreal ranibizumab dose (n = 238) or (3) sham injection cohort (n = 238)		
The 0.5 mg ranibizumab dose was more effective than the 0.3 mg dose, and was thus the dose approved by the Food & Drug Administration and used in the current analysis [18]		
Treatment cohort (0.5 mg ranibizumab) mean vision: MARINA Study for years 1 and 2, then LOCF (last observation carried forward) of clinical trial data for years 3–12 [23]		
Eligible, MARINA sham cohort patients were treated with ranibizumab following the end of the randomized portion of the trial after 24 months. Thus, sham treatment, control cohort data utilized mean vision in the MARINA Study [18] for years 1 and 2, and a Lineweaver–Burke plot meta-analysis control cohort from six randomized, neovascular AMD clinical trials for years 3–12 of sham therapy [25]		
Adverse event disutility QALYs, a total of 0.045 QALY, were used to calculate adverse event QALYs subtracted from total patient value gain		
The average participant received 22 × 0.05 cc intravitreal injections, given approximately monthly, over 2 years		
Mean life expectancy: 12 years for the control and ranibizumab study cohorts [49]		
Value-Based Medicine^©^, base case, cost-utility analysis parameters for the AMD genetic screening, cost-utility analysis [18, 20, 22, 23]		
The patient value and financial value gains are those associated with genetic screening for neovascular AMD making possible the incremental earlier detection and earlier ranibizumab therapy for neovascular AMD		
Model timeline: 12 years = mean life expectancy for average neovascular AMD patient [18]		
Baseline vision in the early-treatment, ranibizumab therapy cohort was 20/40–20/80. Final vision outcome was 20/40^–1^ [20]		
20/40-1 vision in each eye equates with a utility of 0.789 [30, 39]		
Baseline vision in the late-treatment, ranibizumab therapy cohort was 20/160-20/320. Final vision outcome was 20/160^+2^.[20]		
20/160^+2^ vision in each eye equates with a utility of 0.0.658 [29, 30]		
Loss of vision in a first eye results in a utility loss of 0.0398 [35]		
The *incremental* cost-utility analysis per patient utilizes the incremental patient and incremental financial value gains associated with early-treatment ranibizumab therapy (baseline treatment vision 20/40–20/80) for neovascular AMD versus late-treatment ranibizumab therapy (baseline treatment vision of 20/160–20/320) [20]		
The costs of genetic screening were compared with the patient value gains and cost savings conferred by early-treatment ranibizumab therapy (versus late-treatment ranibizumab therapy) made possible by genetic screening		
The direct ophthalmic medical treatment costs were the same in the early-treatment, ranibizumab therapy and late-treatment, ranibizumab therapy cohorts, and therefore were not considered incremental costs		
Only Category 3 AMD eyes in patients who were 65 years of age were tested with genetic screening		
Category 3 AMD cases (drusen >125 μm) annually in the United States in a 65-year-old cohort = 944,400 [1–3]		
22.5% of baseline Category 3 cases have a high-risk genetic profile to develop neovascular AMD [4]		
The phenotypic appearance of Category 3 AMD determines the use of AREDS supplement therapy to decrease the incidence of progression to neovascular AMD [5]. Since the use of AREDS supplements in Categories 1 and 2 AMD has not yet been shown to reduce progression to neovascular AMD [5]. Genetic screening was not presumed to be of benefit to detect whether to use AREDS supplements at an earlier stage than Category 3 AMD		
Genetic testing of Category 4 AMD patients for the development of more severe atrophic changes was not presumed to be of benefit		
Genetic testing was not presumed to be of benefit if one eye was already affected by neovascular AMD or advanced atrophic AMD		
Patients underwent genetic screening at age 65, since only 5.6 % of neovascular AMD develops in patients under the age of 65 years [18]		
Baseline time: First presentation for neovascular AMD occurs at a mean age of 75 years, as per a combination of multiple clinical trials dealing with therapy for neovascular AMD [18–20, 23, 24, 50, 51]		
The outcomes included the QALY (quality-adjusted life-year) gain, percent patient value (quality-of-life) gain, and the CUR (cost-utility ratio), or dollars expended per QALY gained ($/QALY) [22]		
All eyes with neovascular AMD were presumed treated with ranibizumab, including cases presenting with bilateral disease		
Time tradeoff utilities were derived from a database of over 1100 ophthalmic patients with respective levels of vision loss [22–24, 30–38]		
Cost perspectives: societal and 3rd party insurer		
The societal cost perspective included those saved by the better mean vision outcome associated with early-treatment ranibizumab therapy versus late-treatment ranibizumab therapy. They include: (1) direct ophthalmic medical costs = AMD genetic testing costs + incremental annual ophthalmic examination and annual optical coherence tomography costs, (2) direct non-ophthalmic medical costs saved = decreased depression costs, decreased trauma costs, decreased Skilled Nursing Facility costs, decreased nursing costs and other, as yet unidentified, medical costs [40]. (3) direct non-medical costs (caregiver) saved [41] and (4) indirect medical (employment) costs saved [3, 42]		
The 3rd party insurer cost perspective includes: (1) direct ophthalmic medical costs expended and (2) direct non-ophthalmic medical costs saved		
Cost basis: 2012, average, national, Medicare Fee Schedul		
Net present value (NPV) analysis discounted patient value outcomes and costs at a 3 % annual rate. All costs were converted to 2012 US real dollars [22]		
A combined-eye model, a weighted average of first-eye and second-eye models, was utilized to calculate QALY gain per early-treatment case over late-treatment case [23, 24]		
Taking into account the annual conversion rate, the QALY gain and financial value gain accrual rate of 1st eyes with NVAMD was 85.3 % that of 2nd eyes		
Second eyes in the per patient, early treatment benefit from ranibizumab were assumed to have the same visual outcome as treated first eyes		
For the overall Category 3 cohort undergoing genetic testing, the early-treatment ranibizumab QALY gain and costs per person were multiplied by: (Percent of first eyes with presenting vision ≤20/160, or 78.0 %) × (Percent of second eyes with presenting vision ≤20/160 = 62.2 %) × (Sensitivity of genetic testing with phenotypic features for detecting 10-year conversion to NVAMD = 90 %). Thus, overall Category 3 cohort results average (78.0 % × 62.2 % × 90 % =) 43.7 % × (per patient value and financial value outcomes)		
Conversion rates of second eyes to neovaoscular AMD with Markov modeling demonstrated the patient value (QALY gain)		
It was assumed that the majority of patients converted to neovascular in one eye first and thus treated with ranibizumab initially in this first eye, though a small number of cases might have present with bilateral neovascular AMD. This assumption is conservative and biases against the analysis by decreasing the overall patient and financial gains		

*AMD* age-related macular degeneration, *NVAMD* neovascular age related macular degeneration, *QALY* quality-adjusted life-year, *CUR* cost-utility ratio, *MARINA* Minimally classic/occult trial of the Anti-VEGF antibody Ranibizumab In the treatment of Neovascular Age-Related Macular Degeneration, *SNP* single nucleotide polymorphism

### AMD demographics

The AREDS Research Group [5–7] defined four categories of AMD and showed oral supplements decrease the progression of Category 3 AMD (macular drusen ≥125 µm) to NVAMD, though not from Category 3 to central geographic atrophy. Yu and colleagues [4] refined the AREDS four-category model to a five-category model, separating AREDS [5–7] advanced AMD cases into central geographic atrophy (Category 4) and NVAMD (Category 5) in their genotypic/phenotypic study of AMD. Approximately 1.54 million people had NVAMD in the US in 2012 [2, 3]. Central geographic atrophy (AREDS Category 4) in at least one eye was present in 1.24 million, and 8.34 million had drusen ≥125 µm (AREDS Category 3). The 944,000 people aged 65 years with Category 3 drusen (≥125 µm) annually were those who were theoretically screened in our cost-utility model [2, 3].

Fifteen-year, incidence data from the Beaver Dam Study [26] suggest approximately 171,350 new cases of NVAMD develop annually in the US. Among these, 5.6 % in the Minimally classic/occult trial of the Anti-VEGF antibody Ranibizumab In the treatment of Neovascular AMD (MARINA) Study [18, 20] presented before age 65. We excluded that percentage from our analysis since our model assumed genetic testing at age 65, leaving 161,754 annual new cases.

Yu et al. [4], noted 20 % of Category 3 patients progressed to NVAMD (Category 5 AMD) over 10 years (Table 1). They also found 22.5 % (343/1527) of Category 3 patients had a high-risk genetic profile (homozygous for genetic loci on relevant alleles). Among progressors to NVAMD, 90 % had a high-risk genetic profile [4]. Thus, genetic testing for Category 3 AMD patients identified 90 % of progressors to NVAMD, 47 % higher than the 43.0 % identified over 10 years in the Blue Mountains Study [8].

### Utility analysis

The quality-of-life associated with AMD has been quantified using time tradeoff utility analysis [27–37]. Utility anchors are 1.00 (normal bilateral vision permanently) and 0.00 (death). Vision utilities correlate most highly with acuity in the better-seeing eye, rather than the underlying disease [28]. As vision in the better-seeing eye decreases, the associated utility decreases [27–35]. Vision of no light perception bilaterally has an associated utility of 0.26 [30].

Ophthalmic utilities are valid [32], reliable [33], and negligibly affected by systemic comorbidities [34]. The Wills Eye Institute Institutional Review Board approved utility acquisition.

Ophthalmologists underestimated the quality-of-life associated AMD levels by 96–750 % compared to AMD patients, with community utility estimates even more disparate [31]. Thus, VBM cost-utility analyses use patient utilities [22]. Utilities herein were derived from a >1100 direct interview, vision utility database from the Center for Value-Based Medicine^®^ [28–37].

### Value-Based Medicine^®^

Value-Based Medicine^®^ (VBM) integrates the highest level, evidence-based, clinical trial data with standardized inputs, including: (1) time tradeoff utilities, (2) patient utility respondents, (3) a national Medicare Fee Schedule, and (4) societal and 3rd party insurer cost perspectives [22–24]. VBM has been used extensively in ophthalmology, especially for AMD interventions [22–24, 36, 37].

Originated at the Center for Value-Based Medicine^®^ and based upon primary, ophthalmic patient data, the *first*-*eye model* assumes vision loss occurs in one eye, while the fellow eye has good vision [22–24, 36]. In this instance, full patient value gain is not accrued until the fellow eye also develops NVAMD. Utility data from Center for Value-Based Medicine files demonstrate a mean utility difference of 0.0398 between unilateral good vision and 20/40–20/80 vision in the second eye, versus unilateral good vision and ≤20/160 vision in the second eye. This utility gain was weighted to appropriate first-eye model instances herein.

The second-eye model assumes first-eye vision has been lost and the second eye is affected [22–24, 36]. Thus, greater patient value gain occurs with ranibizumab therapy. The combined-eye model used here integrates weighted first-eye model and second-eye models [22, 24]. Extrapolation of data from Barbazetto and colleagues [39] with Markov modeling (TreeAgePro for Healthcare 2012, Williamstown, MA, USA) showed 82 % of patients with unilateral NVAMD in the MARINA/ANCHOR trials developed bilateral NVAMD within 5 years, rising to 96 % by 12 years, the model timeline (Table 3).

**Table 3:** Fellow eye conversion to neovascular AMD in the MARINA/ANCHOR trials, Barbazetto et al. [39]

By end of year	A	B = 46.4 % + (A × 53.5 %)
	Cumulative, incremental, conversion rate of the 53.6 % unilateral, neovascular AMD cases at baseline (%)	Bilateral involvement (%)
Baseline	0.0	46.4
Year 1	21.2	57.7
Year 2	38.0	66.8
Year 3	49.8	73.1
Year 4	59.5	78.3
Year 5	67.2	82.4
Year 6	73.4	85.7
Year 7	78.5	88.5
Year 8	82.6	90.6
Year 9	85.9	92.4
Year 10	88.6	93.9
Year 11	90.7	95.0
Year 12	92.5	96.0
Final	92.5	96.0

Baseline, year 1 and year 2 prevalences are based upon primary data [39], while years 3–12 are based upon the average incidence of conversion to neovascular during years 1 and 2, using a last observation carried forward (LOCF) methodology with Markov modeling

*AMD* age-related macular degeneration

### Patient value gain

Shah and DelPriore [25] modeled the natural course of untreated NVAMD using control cohorts from six randomized, NVAMD trials. In a meta-analysis using Lineweaver–Burke plots, they demonstrated mean vision loss to 20/640 over 8–9 years, after which vision stabilized. Increasing time since NVAMD highly correlated with increasing vision loss.

A double-blind, randomized, clinical trial, MARINA participants had 20/80 baseline vision in both ranibizumab-treatment and sham-treatment cohorts. The 24-month, mean, ranibizumab-treatment cohort vision was 20/63, while mean sham-treatment vision was 20/160^−2^ [18, 20].

Shah and DelPriore data [25] were employed to model MARINA 25–144 month, non-randomized sham results since many sham-treatment patients were allocated to ranibizumab therapy after month 24. Treatment cohort data for months 25–144 were modeled in a LOCF (last observation carried forward) fashion. Twelve years was selected as the model length since this was the average life expectancy of the average NVAMD patient.

An analysis of baseline vision in 50 consecutive Pennsylvania/New Jersey/Delaware patients presenting with first-eye NVAMD in the vitreoretinal practice of author GCB since 2010 was undertaken. The mean, baseline, first-eye vision was 20/182, while that in the fellow eye was 20/47. Overall, 78 % (39/50) presented with vision ≤20/200 in the first eye. Only 18 % (9/50) had first-eye vision ≥20/80 vision at presentation (Table 4).

**Table 4:** Category 3 AMD cases undergoing genetic testing: baseline vision in neovascular AMD eyes in clinical practice integrated with MARINA trial presenting percentages

Model	n	% of baseline patients [18, 20]^a^	Mean vision	% with initial vision 20/40–20/80	% with initial vision ≤20/160
A. 1st eye	50	53.6	20/182	18	78.0
B. 2nd eye	98	46.4	20/84	37	62.2
Both eyes (A × B)	148	100.0	20/136	6.7	48.5
C. Sensitivity of genetic testing	148	90	NA	A × B × C = 6.0 %	A × B × C = 43.7 %

*AMD* age-related macular degeneration, *NA* not applicable

^a^MARINA (Minimally classic/occult trial of the Anti-VEGF antibody Ranibizumab In the treatment of Neovascular AMD) clinical trial [18, 20]

An analysis of 98 consecutive patients presenting with second-eye NVAMD revealed a mean vision of 20/94. Overall, 62.2 % had vision ≤20/160. The vision was ≥20/80 in 37 % of eyes (Table 4).

Critical to our analysis, the excellent report by Boyer et al. [20] demonstrated NVAMD early-treatment eyes (baseline vision 20/40-20/80) had better long-term vision (mean 20/40^−1^) than late-treatment eyes (baseline vision ≤20/160 and mean long-term vision of 20/160^+2^) [20]. Total patient value gains (Table 4), to account for those eyes presenting with ≤20/160 vision and other parameters, had an overall multiplier of 78 % (1st eyes presenting with vision ≤20/160) × 62.2 % (2nd eyes presenting with ≤20/160) × 85.7 % (combined-eye multiplier to account for NVAMD conversion to both eyes) × 90 % (genetic testing sensitivity) = 37.4 %. Adverse events disutilities were subtracted in early-treatment and late-treatment cohorts for months 1–144 [23].

### Costs

The mean, incremental, 12-year, direct, ophthalmic medical costs included genetic testing/monitoring. Ranibizumab therapy costs were excluded since they were assumed similar for early-treatment and late-treatment cohorts, though differences were analyzed in the sensitivity analysis.

Based upon work by Yu et al. [4], genetic testing costs are shown in Table 5. Compounded from $1461 at the time of genetic testing (age 65) to base-case age 75 at the initiation of ranibizumab therapy, they totaled $1906. A $299 cost for an extra, annual, ophthalmic examination and optical coherence tomogram (three/year rather than two/year) was included in the genetic costs for the 22.5 % [4] of genetic-screened high-risk patients progressing to NVAMD. The total cost for genetic testing/monitoring of Stage 3 AMD patients was $2205 per capita.

**Table 5:** Genetic testing costs for genes as per Yu et al. [4]

CPT code	Explanation	Multiplier	NLA	Total NLA
83891	DNA Isolation	1	$5.7	$5.7
83900	Amplification of patient nucleic acid, multiplex, first two nucleic sequences	1	$47.8	$47.8
83901	Amplification of patient nucleic acid, multiplex, each additional acid sequence	10	$23.7	$237
83892	Digestion of amplified DNA with restriction endonuclease enzyme, each	2	$5.7	$11.4
83914	Mutation identification by enzymatic ligation or primer extension, single segment, each segment [e.g. oligonucleotide ligation assay (OLA), single base chain extension (SBCE), or allele-specific primer extension (ASPE)]	15	$23.7	$356
83912	Interpretation and report	1	$5.7	$5.7
83896	Nucleic acid probe, each	15	$5.7	$85
83903	Molecular diagnostics; mutation scanning by physical properties, single segment, each	15	$23.7	$356
83908	Molecular diagnostics; signal amplification of patient nucleic acid, each nucleic acid sequence	15	$23.7	$356
Cost per patient tested				$1461
Adjusted for a genetic testing age of 65 years and ranibizumab. Total treatment age of 75 years (compounded at 3 % annually)				$1906
Including the $299 incremental yearly ophthalmic examination and optical coherence. Total tomogram in the 20 % of patients with a high-risk genetic score [4]				$2205

*CPT* Current Procedural Terminology, published by the American Medical Association, *NLA* National Limitation Amount, all cost in 2012 US nominal dollars

The base-case scenario shows that 30.3 Category 3 AMD patients required screening/monitoring to facilitate one early-treatment. The total cost of screening/monitoring for each early-treatment patient was therefore $66,873 (30.3 × $2205).

Twelve-year negative costs accrued against genetic testing/monitoring costs [4, 20, 39–41]. Costs saved by early-treatment, vs. late-treatment (Table 6), are addressed below [39–41].

**Table 6:** Societal costs (2012 US real dollars) per early-treatment patient from genetic testing/monitoring for neovascular AMD enabling incremental early-treatment (vs. late-treatment) ranibizumab therapy

Societal costs per NVAMD patient treated with intravitreal ranibizumab therapy	Column A 12-year, societal costs integrating 2nd eye multiplier of 85.7 %	Column B 12-year, societal costs adjusted for 1st and 2nd eye = 48.5 % × 90 % gene testing sensitivity^a^	Column C 12-year, direct medical costs, adjusted as in Column B
Direct ophthalmic medical costs (genetic testing/monitoring of patients)	A. $66,873 (per incremental early-treatment ranibizumab case)	A. $66,873 (per incremental early-treatment ranibizumab case)	A. $66,873 (per incremental early-treatment ranibizumab case)
Genetic screening for Category 3 AMD patients with one ranibizumab-facilitated early-treatment case per 30.3 screened cases	$57,805	$57,805	$57,805
1 more ocular (exam/OCT)/year in high-risk genetic cases (22.5 % of Category 3 cases)	$9068	$9068	$9068
Direct non-ophthalmic medical costs [39]	B. (−$93,699)	B. (−$40,914)	B. (−$40,914)
Decreased depression^b^	(−$7169)	(−$3130)	(−$3130)
Decreased injuries^b^	(−$3793)	(−$1656)	(−$1656)
Decreased nursing home admissions^b^	(−$22,351)	(−$9759)	(−$9759)
Less skilled nursing facility (SNF) costs^b^	(−$10,193)	(−$4451)	(−$4451)
Other decreased Medicare costs^b^	(−$50,194)	(−$21,917)	(−$21,917)
Direct non-medical costs (caregiver costs)^b^ [40]	(−$394,929)	(−$172,443)	NA
Indirect medical costs (employment costs)^b^ [41]	(−$32,288)	(−$14,098)	NA
Total negative costs	(−$520,917)	(−$227,455)	(−$40,914)
Total costs	(−$454,044)	(−$160,582)	$25,960

Societal real costs = direct ophthalmic medical costs, direct non-ophthalmic medical costs, direct non-medical costs and indirect medical costs. Cost savings are assumed to accrue beginning at month 7 after the initiation of intravitreal ranibizumab therapy. All costs are discounted at 3 %/year

*NVAMD* neovascular AMD, *OCT* optical coherence tomogram

^a^First eyes: 78 % of first eyes (with initial vision <20/160) adjusted for conversion to NVAMD in 2nd eyes × (85.3 % multiplier); second eyes: 62.2 % with <20/160 vision; 90 % = gene testing/clinical sign sensitivity for detecting future NVAMD

^b^Negative cost

Javitt and colleagues [39] demonstrated increased direct, non-ophthalmic, medical costs for depression, trauma, Skilled Nursing Facilities, nursing homes and unidentified entities associated with vision loss (Table 6). This 12-year cost gained by improving vision per early-treatment patient is (−$40,914) (Table 6).

Schmier and associates [40] reported increasing caregiver costs associated with decreasing levels of vision. Early-treatment ranibizumab therapy resulted in a 12-year, (−$172,443) caregiver cost saving vs. late-treatment therapy (Table 6).

Vision loss decreases employment by 45.6 % and hourly wage loss by 32.5 % referent to age-matched normals, resulting in 36.7 % of normal earnings (Table 6) [41]. Integrating age-related, US employment levels, early-treatment ranibizumab therapy accrued a 12-year employment cost gain of (−$14,098) [41].

## Results

### Patient value gain

The mean MARINA, early-treatment, 20/40^−1^ vision outcome correlated with a 0.789 utility, while late-treatment 20/160^+2^ vision correlated with a 0.658 utility (Table 7) [20, 29, 30]. Additionally, 17.9 % of early-treatment, MARINA eyes achieved vision ≥20/25 bilaterally [20], (utility = 0.97 for bilateral 20/20–20/25 and 0.89 for unilateral 20/20–20/25). This conferred a 12-year, additional 0.121 QALY gain for early-treatment, a 2.0 % quality-of-life gain. First-eye therapy conferred 0.0884 QALY gain, a 1.5 % quality-of-life gain.

**Table 7:** Patient value gain from genetic screening-enabled, early-treatment ranibizumab therapy for neovascular AMD, integrating first-eye and second-eye MARINA Study models

Cohort [18, 20, 23]	Mean vision at 12, 24, and LOCF to 144 months	Utility at 12, 24, and LOCF to 144 months	12-year QALY accrual	Per early-treatment patient QALY gain (QOL gain) integrating conversion of 2nd eyes to NVAMD without adjustments	Per early-treatment patient QALY gain (QOL gain) (adjusting for 1st eye and 2nd eye, 78.0 % × 62.2 %, with 90 % gene testing sensitivity)
12 months Sham 24 months 96 months (baseline vision = 20/40–20/80)	20/126 20/160^−2^ 20/640	0.682 0.657 0.538	5.990	0.0 (0.0 %)	0.0 (0.0 %)
Early ranibizumab treatment (mean baseline vision = 20/40–20/80)	20/40^−1^	0.789	7.924	1.933 (32.8 %)	0.845 (14.1 %)
Late ranibizumab treatment (mean baseline vision = ≤20/160)	20/160^+2^	0.658	6.561	0.571 (9.5 %)	0.250 (4.2 %)
Incremental gain, early vs. late ranibizumab treatment patient made possible by genetic testing	NA	0.141	1.363	1.363 (23.3 %)	0.595 (10.0 %)

Cohort	NA	NA	12-year QALY accrual	Per screened patient QALY gain (QOL gain) integrating 2nd eye conversion to NVAMD	Per screened patient QALY gain (QOL gain) (adjusting for 1st eye and 2nd eye, 78.0 % × 62.2 %, with 90 % gene testing sensitivity)
Incremental gain, per patient genetic screened/monitored for NVAMD	NA	NA	0.0432	0.0432 (0.71 %)	0.0185 (0.33 %)

*LOCF* last observation carried forward, *QOL* quality-of-life, *QALY* quality-adjusted life-year, *NA* not applicable, *NVAMD* neovascular age-related macular degeneration

The 12-year, combined-eye model, QALY accrual for each case of screening-facilitated, early-treatment, ranibizumab therapy was 0.845 QALY, a mean, a 14.1 % quality-of-life gain over sham therapy. Late-treatment accrued 0.250 QALY, a 4.2 % quality-of-life gain over sham-therapy. Thus, early-treatment conferred a, 10.0 %, absolute, quality-of-life gain, a 0.595 QALY gain per early-treatment patient. The QALY gain per genetic screened/monitored patient was 0.0177, a 0.33 % quality-of-life gain. Bilateral good vision QALY gain and first-eye model QALY gain were weighted appropriately

### Costs

Direct ophthalmic medical costs for early-treatment vs. late-treatment ranibizumab therapy were assumed the same, thus excluded in the base case analysis, but addressed in the sensitivity analysis.

Assuming one early-treatment case per 30.3 genetically screened cases, the base-case genetic testing/monitoring cost for each early-treatment case was $66,873 (Table 6).

The incremental negative cost for each early-treatment ranibizumab patient was (−$227,455) (Table 6). With genetic testing/monitoring costs for Category 3 patients screened of $66,873 for each incremental early-treatment case, the overall societal cost per early-treatment case was (−$160,582) (Table 6).

The national, direct ophthalmic cost for genetic testing/monitoring for an annual cohort of 944,400 Category 3 AMD patients million was $2.082 billion. Total negative costs were $7.083 billion. This resulted in a 12-year, financial return-on-investment (ROI) of 240 % referent to genetic testing/monitoring costs. An incremental $260 million societal saving occurred for each 1 % of patients undergoing early-treatment ranibizumab therapy. When genetic testing facilitated an incremental 12,965 (8.0 %) of the 161,754, annual NVAMD patients in the US to undergo early-treatment ranibizumab therapy, an overall, net financial gain for society accrues at the rate of $160,582 per additional early-treatment patient.

The financial ROI distribution, assuming that genetic testing for 30.3 Category 3 AMD cases resulted in one incremental early-treatment case is shown in Table 8. The negative cost for each patient screened (cost of $2205) was (−$7500), an overall societal cost of (−$5295), also a 240 %, 12-year societal ROI. The ROI offset Medicare screening costs by 35 %, Medicaid costs by 63 % and commercial insurer costs by 22–24 %. Patients had the greatest ROI for out-of-pocket genetic testing costs, a net $6725. This converted to a 12-year 16,945 % ROI.

**Table 8:** 12-year return-on-investment (ROI) for early-treatment (per patient screened) for ranibizumab therapy for neovascular AMD

12-year costs (US 2012 real dollars) associated with early-treatment vs. late-treatment ranibizumab therapy	Genetic screening/monitoring costs expended (%) per patient screened	Negative societal costs returned for genetic costs expended (% = percent of total costs returned)	Net final cost	12-year financial return-on-investment (ROI)	Diminution in usual cost due to costs returned from genetic testing
Overall costs	$2205 (100 %)	(−$7500) (100 %)	(−$5295)	240 %	340 %
Medicare (Medicare pays 80 % of Part B costs)	$1559 (70.7 %)	(−$548) (7.3 %)	$1011	−65 %^a^	35 %
Medicaid	$84 (3.8 %)	(−$53) (0.7 %)	$31	−37 %^a^	63 %
Commercial insurance (Medi-gap policies covering 20 % of approved Medicare cost)	$348 (15.8 %)	(−$83) (1.1 %)	$266	−78 %^a^	22 %
Commercial insurance (under age 65 years)	$174 (7.9 %)	(−$53) (0.7 %)	$122	−76 %	24 %
Patients (NB. Healthcare insurance costs are not included)	$40 (1.8 %)	(−$6765) (90.2 %)	(−$6725)	16,945 %	17,045 %

Note the direct ophthalmic medical costs associated with ranibizumab therapy are the same for the early-treatment and late-treatment ranibizumab therapy cohorts, thus not integrated. Costs with parentheses () indicate negative costs that accrue against the direct ophthalmic medical costs expended for ranibizumab therapy

*AMD* age-related macular degeneration

### Cost-utility ratio (CUR)

*$144,000/QALY* For a $144,000/QALY CUR, the upper limit of cost-effectiveness in the US according to World Health Organization criteria [43], an incremental 4.1 % of annual NVAMD patients were required to undergo genetic testing-enabled, early-treatment, ranibizumab therapy. This 4.1 % converts to 6634 patients among the 161,754 annual cohort of new NVAMD patients, 92,200 of whom are not identified as high risk to develop NVAMD by phenotypic features alone (Table 9) [1–3, 9]. It also equates to 1 per 142 of the 944,000 Category 3 AMD patients screened at age 65 annually. For a 3rd party insurer CUR of $144,000/QALY, an increment of 10.1 % of all annual NVAMD patients had to undergo early-treatment for cost-effectiveness (Table 10).

**Table 9:** Societal, cost-utility ratios for category 3 [5] age-related macular degeneration (AMD) patients undergoing genetic testing to predict progression to neovascular AMD

Incremental % of patients undergoing early-treatment ranibizumab therapy due to genetic testing (# of patients)	Genetic testing/moni-toring medical costs^a^ (000s)	Negative costs^a^ (000 s)	Total costs (direct medical + negative costs (000s)	Overall cost (direct medical + negative costs) per patient tested	QALY gain per patient screened	$/QALY
1 % (1618)	$2,082,402	(−$367,918)	$1,714,484	$1815	0.0010	$1,781,400
2 % (3235)	$2,082,402	(−$735,835)	$1,346,567	$1426	0.0020	$699,562
3 % (4853)	$2,082,402	(−$1,103,752)	$978,649	$1036	0.0031	$338,949
4 % (6470)	$2,082,402	(−$1,471,670)	$610,732	$647	0.0041	$158,642
4.1 % (6634)	$2,082,402	(−$1,508,464)	$573,938	$605	0.0042	$144,000
4.5 % (7119)	$2,082,402	(−$1,607,802)	$474,600	$450	0.0045	$100,000
5 % (8088)	$2,082,402	(−$1,839,588)	$242,814	$257	0.0051	$50,458
10 % (16,175)	$2,082,402	(−$3,679,175)	(−$1,596,774)	(−$1691)	0.0102	(−$165,910)
15 % (24,263)	$2,082,402	(−$5,518,763)	(−$3,436,361)	(−$3639)	0.0153	(−$238,032)
20 % (32,351)	$2,082,402	(−$7,358,351)	(−$5,275,949)	(−$5587)	0.0204	(−$274,093)
30 % (48,526)	$2,082,402	(−$11,037,527)	(−$8,955,125)	(−$9482)	0.0306	(−$310,155)
34.6 % (56,048)	$2,082,402	(−$12,738,343)	(−$10,665,141)	(−$11,294)	0.0353	(−$319,834)
38.5 % (62,275)	$2,082,402	(−$14,164,826)	(−$12,082,424)	(−$12,794)	0.0392	(−$326,078)

The societal costs perspective included the following costs: (a) direct ophthalmic medical costs, (b) direct non-ophthalmic medical costs (depression, trauma, skilled nursing facility, nursing home, other Medicare costs) + caregiver costs + employment costs saved by genetic testing leading to early-treatment, versus late-treatment, of neovascular age-related macular degeneration with intravitreal ranibizumab

Direct non-ophthalmic medical costs = costs for depression, trauma, skilled nursing facilities, nursing homes and other Medicare costs

The model is a combined-eye model integrating the patient value gain and costs associated with first-eye (78.0 % presenting with vision <20/160) and second-eye (62.2 % of eyes presenting with 20/20/160 vision) ranibizumab therapy for neovascular AMD, assuming genetic testing identifies 90 % of cases that will progress to neovascular age-related macular degeneration

A negative cost-utility ratio () indicates that early-treatment dominates late treatment, meaning that early-treatment accrues greater QALYs and has a positive financial return-on-investment

*QALY* quality-adjusted life-year, *$/QALY* cost-utility ratio, or dollars expended/gained per QALY gained from genetic testing

^a^Direct ophthalmic medical costs = costs associated with genetic screening, including $1906 for screening and $299 for 1 extra annual examination and optical coherence tomography scan for the 22.5 % of Category 3 AMD patients with a high risk genetic profile for progression to neovascular age-related macular degeneration

**Table 10:** 3rd party insurer, cost-utility ratios for Category 3 [5] age-related macular degeneration patients undergoing genetic testing for progression to neovascular AMD

Incremental % of patients undergoing early-treatment ranibizumab therapy due to genetic testing (# of patients)	Genetic testing/moni-toring medical costs^a^ (000s)	Negative costs^a^ (000s)	Total costs (direct medical + negative costs (000s)	Overall cost (direct medical + negative costs) per patient tested	QALY gain per patient screened	$/QALY
1 % (1618)	$2,082,402	(−$66,180)	$2,016,222	$2135	0.0010	$2,094,915
2 % (3235)	$2,082,402	(−$132,360)	$1,950,042	$2065	0.0020	$1,013,076
3 % (4853)	$2,082,402	(−$198,540)	$1,883,862	$1995	0.0031	$652,463
4 % (6479)	$2,082,402	(−$264,720)	$1,817,682	$1925	0.0041	$472,156
5 % (8088)	$2,082,402	(−$330,900)	$1,751,502	$1855	0.0051	$363,973
10 % (16,175)	$2,082,402	(−$661,800)	$1,420,602	$1504	0.0102	$147,605
10.1 % (16,504)	$2,082,402	(−$675,036)	$1,407,366	$1479	0.0103	$144,000
13.2 % (21,358)	$2,082,402	(−$878,341)	$1,204,061	$1350	0.0135	$100,000
15 % (24,263)	$2,082,402	(−$992,700)	$1,089,702	$1154	0.0153	$75,482
20 % (32,351)	$2,082,402	(−$1,323,600)	$758,801	$803	0.0204	$39,421
30 % (48,526)	$2,082,402	(−$1,985,400)	$97,001	$103	0.0306	$3,360
34.6 % (56,048)	$2,082,402	(−$2,293,138)	(−$210,736)	(−$223)	0.0353	(−$6,319)
38.5 % (62,275)	$2,082,402	(−$2,547,931)	(−$465,529)	(−$493)	0.0392	(−$12,564)

The societal costs perspective included the following costs: (a) direct ophthalmic medical costs, (b) direct non-ophthalmic medical costs (depression, trauma, skilled nursing facility, nursing home, other Medicare costs) + caregiver costs + employment costs saved by genetic testing leading to early-treatment, versus late-treatment, of neovascular age-related macular degeneration with intravitreal ranibizumab

Direct non-ophthalmic medical costs = costs for depression, trauma, skilled nursing facilities, nursing homes and other Medicare costs

The model is a combined-eye model integrating the patient value gain and costs associated with first-eye (78.0 % presenting with vision <20/160) and second-eye (62.2 % of eyes presenting with 20/20/160 vision) ranibizumab therapy for neovascular AMD, assuming genetic testing identifies 90 % of cases that will progress to neovascular age-related macular degeneration

A negative cost-utility ratio () indicates that early-treatment dominates late treatment, meaning that early-treatment accrues greater QALYs and has a positive financial return-on-investment (*QALY* quality-adjusted life-year, *$/QALY* cost-utility ratio, or dollars expended/gained per QALY gained from genetic testing, *AMD* age-related macular degeneration)

^a^Direct ophthalmic medical costs = costs associated with genetic screening, including $1906 for screening and $299 for 1 extra annual examination and optical coherence tomography scan for the 22.5 % of Category 3 AMD patients with a high risk genetic profile for progression to neovascular age-related macular degeneration

For a $100,000/QALY CUR, the upper limit of cost-effectiveness commonly utilized in the US [22], an incremental 4.5 % of annual NVAMD patients were required to undergo early-treatment, ranibizumab therapy for cost-effectiveness (Table 9). For a 3rd party insurer CUR of $100,000/QALY, an incremental 13.2 % of all annual NVAMD patients had to undergo early-treatment for cost-effectiveness (Table 10).

### Sensitivity analysis

Sensitivity analysis (Table 11) showed decreasing age made genetic testing considerably less cost-effective (age 40 societal CUR = $1,088,253/QALY) and 3rd party insurer CUR = $231,137/QALY). Deleting extra ophthalmic monitoring costs had negligible effect, while decreasing genetic testing price improved cost-effectiveness. If the cost of ranibizumab therapy for early-treatment cases is twice that of the ranibizumab cost for late-treatment cases, an increment of approximately 6.0 % (9700) of NVAMD cases undergoing early-treatment ranibizumab therapy is required for genetic testing to be cost-effective using WHO criteria.

**Table 11:** Sensitivity analysis

Cost perspec- tive	Incremental % of patients undergoing early-treatment ranibizumab therapy due to genetic testing (# of patients)	Genetic testing/monitoring medical costs^a^ (000s)	Negative costs (000s)	Total costs (direct medical + negative costs (000s)	Overall cost (direct medical + negative costs per patient tested	QALY gain per patient	$/QALY
$500 cost for genetic testing, screening at age 65							
Societal	4.1 % (6,634)	$754,576	(−$1,508,464)	(−$839,978)	(−$890)	0.0043	(−$321,506)
3rd party	10.1 % (16,504)	$754,576	(−$675,036)	(−$79,540)	(−$84)	0.0103	(−$26,389)
$1000 cost for genetic testing, screening at age 65							
Societal	4.1 % (6634)	$1,226,776	(−$1,508,464)	(−$281,688)	(−$298)	0.0042	(−$71,047)
3rd party	10.1 % (16,504)	$1,226,776	(−$675,036)	$551,740	$584	0.0103	$56,744
Patient value unchanged, change age of screening from 65 to 60 years							
Societal	4.1 % (6634)	$2,817,145	(−$1,508,464)	$1,312,591	$1390	0.0042	$330,075
3rd party	10.1 % (16,504)	$2,817,145	(−$675,036)	$2,142,109	$2,269	0.0103	$220,291
Patient value unchanged, change age of screening from 65 to 52 years							
Societal	4.1 % (6634)	$3,787,988	(−$1,508,464)	$2,279,524	$2415	0.0042	$574,940
3rd party	10.1 % (16,504)	$3,787,988	(−$675,036)	$3,112,952	$3,298	0.0103	$320,167
Patient value unchanged, change age of screening from 65 to 40 year							
Societal	4.1 % (6634)	$5,823,170	(−$1,508,464)	$4.314,706	$4571	0.0042	$1,088,253
3rd party	10.1 % (16,504)	$5,823,170	(−$675,036)	$5,148,134	$5454	0.0103	$529,469
No extra eye exams and OCT in addition to two annual visits, screening at age 65							
Societal	4.1 % (6634)	$1,800,026	(−$1,508,464)	$291,562	$309	0.0042	$73,538
3rd party	10.1 % (16,504)	$1,800,026	(−$675,036)	$1,124,990	$1192	0.0103	$115,702
Four extra eye exams and OCT’s in addition to two annual visits, screening at age 65							
Societal	4.1 % (6634)	$3,211,904	(−$1,508,464)	$1,703,440	$1804	0.0042	$429,641
3rd party	10.1 % (16,504)	$3,211,904	(−$675,036)	$2,536,868	$2687	0.0103	$250,909
Early-treatment ranibizumab cases receive twice as many injections over the first 2 years of therapy than late treatment casers, screening at age 65							
Societal	4.1 % (6634)	$2,667,392	(−$1,508,464)	$1,072,038	$1136	0.0042	$270,591
3rd party	10.1 % (16,504)	$2,667,392	(−$675,036)	$1,992,356	$2111	0.0103	$204,907

Cost-utility (cost-effectiveness) ratios for Category 3 AMD patients screened for neovascular AMD resulting in incremental, early-treatment ranibizumab therapy (2012 US real dollars, combined-eye model [18–20, 22])

A negative cost-utility ratio (parentheses) indicates that early-treatment dominates late treatment, meaning that early-treatment accrues greater QALYs and has a positive financial return-on-investment

*NVAMD* neovascular Age-related macular degeneration, *QALY* quality-adjusted life-year, *$/QALY* cost-utility ratio, or dollars expended/gained per QALY gained from genetic testing, *US* United States informal upper limit for cost-effectiveness, *WHO* World Health Organization upper limit for cost-effectiveness = 3× Gross Domestic Product per capita, *AMD* age-related macular degeneration, *3rd party* third party insurer cost perspective, integrating all incremental direct medical costs associated with genetic screening for neovascular AMD, *Societal* societal cost perspective, including all direct ophthalmic medical, direct non-ophthalmic medical, direct non-medical (caregiver) and indirect medical (employment) costs

^a^Costs associated with genetic screening, =$1906 for screening for each Category 3 AMD patient and $299 for 1 extra annual examination and optical coherence tomography scan for the 22 Category 3 AMD patients with a high risk genetic profile

## Discussion

Our analysis demonstrated, using MARINA Trial data [20], that each incremental, early-treatment, ranibizumab case of NVAMD conferred a combined-eye model, 14.1 % quality-of-life improvement, versus a 4.2 % quality-of-life improvement with late-treatment (baseline vision of 20/160–20/320) (Table 7), a 10.0 %, incremental, patient value gain. The net, societal, financial ROI was $160,582 per early-treatment case, a net $5295 ROI per patient screened.

While the US has no formal, cost-effectiveness upper limits, interventions costing ≤$100,000/QALY are generally believed cost-effective [22]. Formal World Health Organization standards indicate interventions costing ≤3× GDP per capita (~US $144,000) per DALY (disability-adjusted life-year), a metric similar to the QALY, are cost-effective (Tables 9, 10) [42]. Screening is still cost-effective if only 4.1 % overall NVAMD cases, or 7.2 % of cases not forecast phenotypically, receive genetic testing-facilitated early-treatment ranibizumab therapy

The presence of AMD, even with good vision, can decrease a patient’s quality-of-life [31]. We believe low-risk/medium-risk genetic profiles for progression to advanced AMD, likely allay patient fears and improve patient quality-of-life. We are uncertain how a positive, high-risk genetic profile affects patient quality-of-life, but since only 22.5 % of screened patients have a high-risk profile [4], the overall quality-of-life gain in the low-risk and medium-risk genetic profile cohorts could possibly outweigh total quality-of-life loss in the high-risk profile cohort.

### Wealth of the nation

The data herein support the work of Nordhaus [43], the Yale economist who estimated 50 % of the wealth of the United States created during the twentieth century occurred from healthcare advances. While secondary to patient value gain, the increase in national wealth associated with medical interventions is an important factor. If 12,965 (8.0 %) of the 62,279 NVAMD cohort patients not predicted phenotypically to develop NVAMD are recruited to early-treatment due to genetic testing, the cost of testing is at a breakeven point. Each patient undergoing early-treatment in addition adds $160,582 to societal wealth.

### When to genetically screen

We selected the age of 65 years as the most cost-effective age for genetic screening since it encompasses 94.4 % of those who develop NVAMD [18]. As sensitivity analysis shows, the earlier genetic testing is performed, the greater the expense of testing, since analyses must account for the time value of money.

Patients with NVAMD may not come in promptly due to numerous reasons, including: (1) unawareness of NVAMD symptoms [44], (2) not realizing vision is decreased in one eye, (3) denial, (4) absence of pain, (5) believing refraction or cataract is their problem, (6) cognitive difficulties and (7) others. In non-ophthalmic specialties, well-defined follow-up plans [45], good relationships with healthcare providers [46], and focused programs [46] result in greater patient adherence. While we cannot be certain, we are hopeful that high-risk phenotypic/genotypic profile patients will be especially aware to present promptly when they develop NVAMD symptoms.

To our knowledge, convincing data that show more frequent ophthalmic screening allows earlier NVAMD treatment are lacking. Nonetheless, we included the extra costs of screening herein to be conservative in our economic analysis.

Some might argue genetic testing of Category 3 AMD cases is of no benefit over phenotypic progression parameters. While the case for Category 4 atrophic AMD centrally or unilateral NVAMD, phenotypic progression parameters for Category 3 AMD are less reliable [6, 47]. Furthermore, only a small increment (4.1 %) in genetic testing-enabled, early-treatment, ranibizumab therapy cases is necessary for screening cost-effectiveness. As genetic testing likely increases in accuracy and decreases in price, the patient and financial value gains will be more pronounced. Though the study was performed in US dollars, WHO criteria are very similar for many developed countries globally [48].

## Conclusions

In summary, genetic screening for NVAMD is cost-effective by the WHO standard of $144,000/QALY if it facilitates early-treatment with ranibizumab for an incremental 4.1 % of annual NVAMD cases. If genetic testing allows earlier ranibizumab therapy for an incremental one NVAMD case per 142 Category 3 AMD patients screened, it remains cost-effective by WHO standards. Using the often accepted US upper limit of cost-effectiveness of $100,000/QALY, genetic testing for NVAMD is cost-effective if it facilitates an incremental 4.5 % of annual NVAMD cases to undergo early-treatment ranibizumab therapy. This information can be used by clinicians to decide whether genetic testing for neovascular age-related macular degeneration is appropriate for their patients.

## Abbreviations

AMD: age-related macular degeneration
NVAMD: neovascular age-related macular degeneration
AREDS: Age Related Eye Diseases Study
QALY: quality-adjusted life-year
$QALY: dollars expended per quality-adjusted life-year gained
VBM: Value-Based Medicine
CUR: cost-utility ratio
